# Imaging biomarkers of glymphatic system dysfunction in Alzheimer’s disease: a systematic review and meta-analysis with a focus on DTI-ALPS index

**DOI:** 10.3389/fnagi.2026.1749316

**Published:** 2026-06-01

**Authors:** Zulin Liao, Rui Chen, Xinyi Yang, Kailai Shen, Jingjuan Zeng, Haijin Zhou, Chen Fang, Yudan Liu, Chunmei Chen

**Affiliations:** 1 Academy of Integrative Medicine, College of Integrative Medicine, Fujian University of Traditional Chinese Medicine, Fuzhou, China; 2 Fujian-Taiwan Science and Technology Cooperation Base for Integrated Traditional Chinese and Western Medicine Prevention and Treatment of Cardiovascular and Cerebrovascular Diseases, Fujian Province, Fuzhou, China; 3 Second Clinical Medical College, Fujian University of Traditional Chinese Medicine, Fuzhou, China; 4 Department of Neurosurgery, The Second Affiliated Hospital of Fujian University of Traditional Chinese Medicine, Fuzhou, China

**Keywords:** ALPS index, Alzheimer’s disease, biomarker, diffusion tensor imaging, glymphatic system, meta-analysis, mild cognitive impairment

## Abstract

**Background:**

Alzheimer’s disease (AD) has been linked to impaired clearance of metabolic waste, and glymphatic dysfunction is increasingly considered a potential contributor to its pathogenesis. The diffusion tensor imaging-based analysis along the perivascular space (ALPS) index has been proposed as a non-invasive imaging marker, although findings across clinical studies remain inconsistent.

**Methods:**

We systematically searched PubMed, Embase, Web of Science, Scopus, CENTRAL, and PEDro up to August 2025 in accordance with PRISMA guidelines. Studies reporting ALPS index values in adults with AD, mild cognitive impairment (MCI), or cognitively normal controls (NC) were included. Risk of bias was assessed using the AHRQ checklist, and the certainty of evidence was evaluated with GRADE.

**Results:**

Fifteen studies involving 1,756 participants were included in the meta-analysis. Pooled results showed a stepwise decrease in ALPS values, with significantly lower values in AD compared with NC (mean difference −0.20, *I*^2^ = 93%) and MCI (−0.09, *I*^2^ = 78%), as well as in MCI compared with NC (−0.11, *I*^2^ = 92%). Subgroup and sensitivity analyses supported the stability of these findings despite methodological heterogeneity.

**Conclusion:**

The ALPS index shows a progressive decrease across the AD continuum, which is consistent with the presence of glymphatic alterations during disease progression. As a non-invasive MRI-derived marker, ALPS may have potential for use in early detection and monitoring; however, further validation with standardized imaging protocols and longitudinal studies is required before clinical application.

**Systematic review registration:**

https://www.crd.york.ac.uk/PROSPERO/view/CRD420251119624, PROSPERO, CRD420251119624.

## Introduction

Alzheimer’s disease (AD) is the most common neurodegenerative disorder, currently affecting over 50 million people worldwide, with projections exceeding 150 million by 2050 (Jiao H. S. et al., 2025). It is characterized by progressive cognitive decline and loss of independence, placing a substantial burden on individuals and healthcare systems (Mello-Sampayo et al., 2025). Despite extensive research, the underlying mechanisms of AD remain incompletely understood, and effective disease-modifying therapies are still limited.

To explore imaging correlates potentially related to glymphatic function in humans, diffusion tensor imaging (DTI) has been increasingly used as a non-invasive approach. In particular, the analysis along the perivascular space (ALPS) index—derived from diffusivity along projection and association fibers adjacent to the lateral ventricles—has been proposed as a potential imaging marker of glymphatic function (Han et al., 2023; Liu et al., 2025; Satpathi et al., 2025). Several clinical studies have reported reduced ALPS values in patients with AD compared with cognitively normal controls, as well as associations with cognitive performance, PET imaging, and CSF biomarkers (Chen et al., 2025; Liang et al., 2023). However, substantial variability in imaging protocols, sample sizes, and study populations has resulted in heterogeneous and sometimes inconsistent findings.

Given these uncertainties, a systematic review and meta-analysis was conducted to synthesize the available evidence. Specifically, this study aimed to: (1) evaluate alterations in the DTI-ALPS index across AD and related cognitive states (mild cognitive impairment [MCI] and cognitively normal [NC]); (2) quantify effect sizes between groups; and (3) explore potential sources of heterogeneity. Through this approach, we aimed to better characterize ALPS alterations across the AD continuum and to examine its potential relevance as an imaging marker, primarily in research settings, while acknowledging that its clinical applicability remains to be established.

## Method

### Search strategy

This systematic review was conducted in accordance with the Preferred Reporting Items for Systematic Reviews and Meta-Analyses (PRISMA) guidelines. The review protocol was prospectively registered in the International Prospective Register of Systematic Reviews (PROSPERO, CRD420251119624). Literature searches were performed in the following electronic databases: Embase, Web of Science, Scopus, Medline, Physiotherapy Evidence Database (PEDro), PubMed, and the Cochrane Central Register of Controlled Trials (CENTRAL). The following search terms were applied in combination with Boolean operators: (Alzheimer Disease OR Alzheimer’s) AND (Glymphatic System OR Glymphatic Clearance System OR Glymphatic Pathway) AND (Diffusion Tensor Imaging OR DTI OR DTI-ALPS OR ALPS index). The search was restricted to full-text cross-sectional studies, with no date or language limitations.

### Eligibility criteria

The inclusion criteria were defined using the PICOS framework: (1) Population: adults diagnosed with Alzheimer’s disease (AD), mild cognitive impairment (MCI), or cognitively normal (NC); (2) Intervention/Exposure: ALPS index and related imaging parameters derived from diffusion tensor imaging (DTI); (3) Comparison: AD versus MCI or NC, and MCI versus NC; (4) Outcomes: imaging measures related to glymphatic function, particularly mean and standard deviation of the DTI-ALPS index, as well as associations with cognition or other biomarkers; (5) Study design: cross-sectional or case–control studies providing sufficient data for quantitative analysis. Studies including AD, MCI, and other pathological groups without subgroup-specific data were excluded.

### Study selection and data extraction

The literature search was performed using the above databases and keywords, and duplicate records were removed. Titles and abstracts were screened to exclude studies that did not meet the inclusion criteria. Full texts of the remaining studies were then reviewed in detail, and eligible studies were included in the final analysis. Two reviewers (ZLL and RC) independently performed study screening, quality assessment, and data extraction using standardized data extraction forms. Any disagreements were resolved through discussion with a third reviewer (XYY). Extracted data included: (1) author and year of publication; (2) number of participants, age, and group allocation (AD, MCI, NC); (3) imaging characteristics (MRI system, acquisition parameters, regions of interest, main indices); and (4) study outcomes (ALPS index and related imaging or clinical findings). All data were extracted from the article text, tables, and figures. For missing data or additional information, the corresponding authors were contacted.

### Risk of bias assessment

Risk of bias was assessed using the 11-item checklist developed by the Agency for Healthcare Research and Quality (AHRQ). Each item was scored as “yes” (1 point) or “no/unclear” (0 points), with total scores ranging from 0 to 11. Based on the overall score, studies were categorized as low, moderate, or high risk of bias. Two reviewers independently conducted the assessment, and any disagreements were resolved through discussion with a third reviewer.

### Statistical analysis

Subgroup analyses were conducted to explore potential sources of heterogeneity. Studies were categorized into “clinical validation” and “mechanistic exploration” subgroups based on their primary research objectives. Clinical validation studies were defined as those primarily aiming to assess the diagnostic or clinical relevance of the ALPS index in differentiating patient groups or predicting clinical outcomes. In contrast, mechanistic exploration studies focused on investigating the relationships between ALPS and other imaging, molecular, or physiological markers (e.g., PET biomarkers, CSF indicators, or microstructural features), with the aim of providing insight into potential underlying pathophysiological processes. This classification was intended to distinguish studies with different analytical purposes and methodological emphases, thereby providing a structured framework for exploring heterogeneity, rather than implying a strict or exhaustive categorization.

Meta-analysis was performed to compare ALPS values across different populations. Studies were stratified into subgroups based on the reported comparisons (e.g., AD vs. NC, AD vs. MCI, MCI vs. NC). When a single study reported multiple comparisons, each comparison was included in the corresponding subgroup. Mean difference (MD) with 95% confidence intervals (CIs) was used as the effect size, with statistical significance set at *p* < 0.05.

Heterogeneity was assessed using the Chi-square test and the *I*^2^ statistic, with *I*^2^ representing the proportion of total variation attributable to between-study heterogeneity. A fixed-effects model was applied when heterogeneity was low (*I*^2^ ≤ 50%), whereas a random-effects model was used when heterogeneity was high (*I*^2^ > 50%). All analyses were conducted using RevMan software (version 5.4), and results were presented in tables and forest plots.

## Results

**Figure 1:**
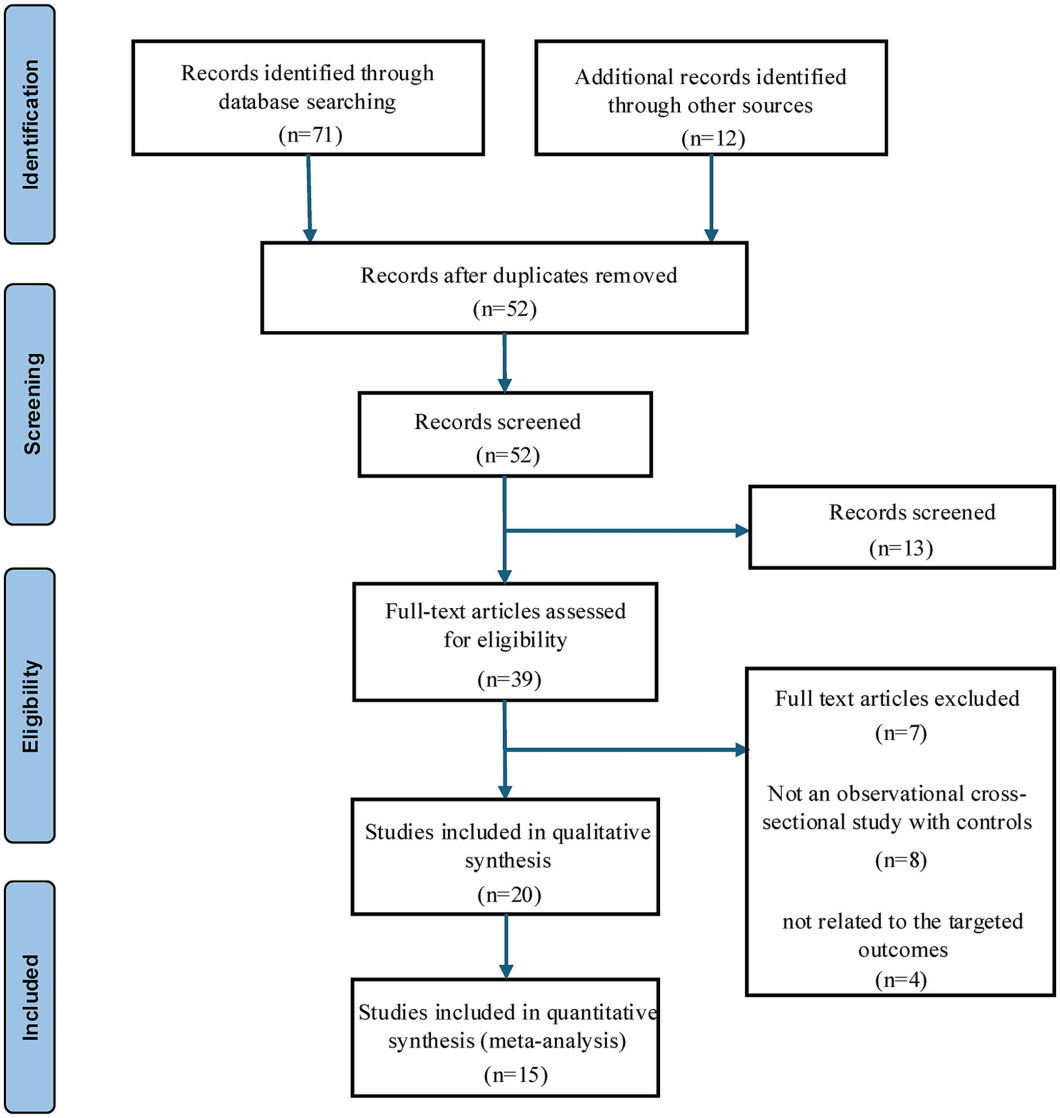
PRISMA flow diagram of study selection.

As shown in Figure 1, a total of 52 potentially relevant articles were identified through database searches. After screening, 20 studies were included in the systematic review, of which 15 were eligible for quantitative synthesis in the meta-analysis.

### Risk of bias assessment

The AHRQ scores of the included studies were generally moderate to high (range: 6–9). Studies by Huang et al. (2024), Sacchi et al. (2024), and Zhang et al. (2025) achieved relatively high scores, indicating more complete reporting and a lower risk of bias. In contrast, several studies showed limitations in reporting data sources and handling missing data. Overall, most studies adequately described participant characteristics and outcome assessments; however, “handling of missing data” and “response rate” remained common limitations (see Supplementary Table S1).

### Study characteristics

In total, 15 studies comprising 1,756 participants (AD = 712, MCI = 462, NC = 582) were included. The largest sample size was reported by Huang et al. (2024) (*n* = 419), whereas the smallest was by Sun et al. (2024) (*n* = 41). Mean participant ages ranged from 59.4 to 78.3 years (Jiao Y. et al., 2025; Kim et al., 2024). Some studies included only AD or MCI patients (Sun et al., 2024; Kim et al., 2024; Shang et al., 2024; Hsu et al., 2023; Ota et al., 2022), while others included both groups (Chen et al., 2025; Liang et al., 2023; Huang et al., 2024; Sacchi et al., 2024; Zhang et al., 2025; Jiao Y. et al., 2025; Chang et al., 2023; Guo et al., 2025; Kamagata et al., 2022; Nakaya et al., 2025; Zhang et al., 2024). Definitions of control groups varied across studies: some used strictly cognitively normal individuals, whereas others included amyloid-*β*–negative older adults (Nakaya et al., 2025). These individuals were generally considered cognitively normal based on the original study definitions, although subtle cognitive changes cannot be entirely excluded. All studies employed MRI to compute the DTI-ALPS index (See Table 1).

**Table 1:** Participant characteristics.

Study (First author, Year)	Cohort/Database	Disease group	*n*	Age, mean ± SD (years)	Sex (M/F)	ALPS index		Control group type	Comparison type	AHRQ score (0–11)
						Mean	SD			
Zhang et al. (2024)	CANDOR cohort	NC	26	63.4 ± 7.4	11/15	1.57	0.16	NC	NC vs. AD-D	8
		AD-MCI	15	66.0 ± 8.6	7/8	1.48	0.13			
		AD-D	15	66.9 ± 9.5	9/6	1.42	0.16			
Kamagata et al. (2022)	ADNI-2	HC	31	73.86 ± 4.91	14/17	1.33	0.08	HC	HC vs. MCI; HC vs. AD; MCI vs. AD	8
		MCI	44	73.38 ± 5.68	26/18	1.33	0.11			
		AD	36	74.28 ± 8.76	22/14	1.21	0.14			
Huang et al. (2024)	ADNI	CN	235	68.7 ± 3.85	85/150	1.28	0.187	CN	CN vs. MCI vs. AD	9
		MCI	137	68.2 ± 5.17	83/54	1.24	0.198			
		AD	47	68.0 ± 5.36	22/25	1.17	0.143			
Chen et al. (2025)	Zhejiang Provincial People’s Hospital memory clinic and Hospital Health Promotion Center	AD	89	68.75 ± 9.29	40/49	1.308	0.015	NC	AD vs. NC, aMCI vs. NC, AD vs. aMCI	8
		aMCI	24	67.25 ± 9.32	12/12	1.45	0.04			
		NC	32	67.41 ± 7.55	12/20	1.57	0.027			
Nakaya et al. (2025)	ADNI-2	AD	26	73.2 ± 9.23	16/10	1.2	0.145	Aβ − HC	Aβ − HC, Aβ + HC, Aβ − SCD, Aβ + SCD, MCI, AD	6
		MCI	19	74.4 ± 4.28	11/8	1.33	0.169			
		Aβ + SCD	9	73 ± 3.8	1/8	1.39	0.128			
		Aβ − SCD	21	72.2 ± 5.25	9/12	1.55	0.21			
		Aβ + HC	10	74.8 ± 4.03	2/8	1.32	0.06			
		Aβ − HC	21	71.6 ± 6.15	10/11	1.61	0.2			
Jiao H. S. et al. (2025)	Single-center hospital-based cohort (First Affiliated Hospital of Harbin Medical University, China)	HC	19	59.4 ± 7.21	4/15	1.286	0.15	HC	AD vs. PAD vs. HC	8
		PAD	16	61.6 ± 5.73	7/9	1.188	0.121			
		AD	18	67.3 ± 8.44	4/14	1.107	0.123			
Guo et al. (2025)	ADNI	CN	35	73 (60–89)	17/18	1.338	0.094	CN	CN vs. SMC vs. MCI vs. AD	8
		SMC	28	73.5 (66–83)	10/18	1.249	0.071			
		MCI	82	74 (55–88)	53/29	1.244	0.068			
		AD	35	75 (64–90)	23/12	1.174	0.077			
	CTPCS-HEAD	CN	25	68 (55–81)	12/13	1.333	0.045			
		SMC	51	68 (55–78)	15/36	1.343	0.049			
		MCI	32	71 (58–81)	15/17	1.312	0.057			
		AD	19	74 (55–90)	8/11	1.222	0.051			
Shang et al. (2024)	Suzhou University Affiliated First Hospital	ADSD	40	68.63 ± 6.19	14/26	1.268	0.069	HC	ADSD vs. NC; ADNSD vs. NC	8
		ADNSD	39	69.90 ± 6.28	16/23	1.363	0.049			
		NC	25	67.36 ± 7.13	12/13	1.586	0.048			
Sun et al. (2024)	Neuro-Memory Clinic, Guizhou Medical University Affiliated Hospital, China	HC	18	63.44 ± 6.92	9/9	1.357	0.127	HC	MCI vs. HC	7
		MCI	23	67.74 ± 6.99	8/15	1.234	0.136			
Sacchi et al. (2024)	Fondazione IRCCS Ca′ Granda Ospedale Maggiore Policlinico, Milan, Italy	nAD	24	75 (70.75, 77.25)	9/15	1.224	0.082	CU	AD vs. CU; MCI vs. CU; nAD vs. CU; AD vs. MCI; AD vs. nAD; MCI vs. nAD	9
		AD	47	71 (67.5, 76)	22/25	1.189	0.097			
		MCI	17	74 (69, 76)	8/9	1.325	0.119			
		CU	23	70 (60, 75.5)	14/9	1.353	0.174			
Hsu et al. (2023)	Chang Gung Memorial Hospital, Taiwan	NCs	13	61.0 ± 7.1	4/9	1.5	0.1	NCs	AD vs. NC	8
		AD	37	63.2 ± 4.7	10/27	1.4	0.2			
Liang et al. (2023)	China–Japan Friendship Hospital	NC	28	67.75 ± 6.09	11/17	1.44	0.07	NC	AD vs. NC; MCI vs. NC; VCI vs. NC	8
		AD	38	72.05 ± 6.95	10/28	1.15	0.07			
		MCI	18	71.28 ± 8.66	9/9	1.19	0.07			
		VCI	21	69.33 ± 6.37	9/12	1.21	0.05			
Ota et al. (2022)	Single-center, prospectively recruited participants (Japan)	AD	21	71.3 ± 9.1	7/14	1.18	0.15	Healthy subjects	AD vs. Hs	8
		HS	36	64.6 ± 7.6	15/21	1.4	0.18			
Zhang et al. (2025)	—	AD	140	70.00 [64.00, 74.00]	34/65	1.32	0.14	SCD	AD vs. MCI vs. SCD	9
		MCI	35	65.74 ± 9.06	19/16	1.37	0.13			
		SCD	6	59.50 ± 6.68	3/3	1.51	0.08			
Kim et al. (2024)	Dementia Clinic, Seoul National University Bundang Hospital, Republic of Korea	AD	65	78.3 ± 8.3	17/53	1.476	0.081	CN	AD vs. CN	8
		CN	15	70.7 ± 6.2	8/7	1.784	0.168			

### Imaging characteristics

To facilitate interpretation of methodological variability, diffusion MRI acquisition parameters across included studies were summarized (Table 2). All studies employed 3T MRI systems to derive the ALPS index. Diffusion weighting was most commonly performed with *b*-values of 1,000 s/mm^2^, although some studies used multi-shell acquisitions (e.g., *b* = 1,000 and 2,000 s/mm^2^). The number of diffusion gradient directions varied substantially across studies, ranging from 25 to 64 directions. Spatial resolution also differed between studies, with voxel sizes typically around 2 mm isotropic in some studies, while higher-resolution acquisitions were reported in others. In terms of ROI definition, most studies focused on projection and association fibers adjacent to the lateral ventricles, although several extended analyses to additional regions such as the basal ganglia, hippocampus, or centrum semiovale. Preprocessing pipelines were heterogeneous, including different software platforms and correction procedures (e.g., FSL, ANTs, Dipy, TORTOISE, and deep learning-based methods). These variations in acquisition and analysis protocols may contribute to between-study heterogeneity in ALPS measurements.

**Table 2:** Summary of imaging characteristics, methodological features, and key findings of the included studies.

Study (First author, Year)	Imaging modality	Sequence/parameters	ROI	Main imaging index	Key findings
Zhang et al. (2024)	MRI (DTI-ALPS, T1w, T2WI, FLAIR)	3 T Siemens Prisma; DTI, T1-MPRAGE, T2WI, T2-FLAIR; preprocessing with TORTOISE and ANTs	Projection and association fibers near lateral ventricle body (LVB); PVS measured at basal ganglia (BG), centrum semiovale (CSO), and LVB	ALPS index; PVS volume fraction	AD-D had ↓ ALPS and ↑ BG-PVS vs. NC; lower ALPS correlated with worse cognition.
Kamagata et al. (2022)	MRI (DTI, structural T1w, FLAIR), PET (Aβ, FDG), CSF biomarkers	3 T MRI; DTI; T1w and FLAIR; preprocessing with FSL/Dipy; PVS mapping and FW estimation	White matter (centrum semiovale), basal ganglia, hippocampus; deep medullary veins for ALPS	ALPS index, PVS volume fraction (PVSVF), free-water fraction in WM (FW-WM)	AD showed ↑ PVSVF, ↑ FW-WM, ↓ ALPS; MCI had preserved ALPS; low ALPS linked to ↓ Aβ42, ↓ FDG uptake, and cognitive decline.
Huang et al. (2024)	MRI (DTI-ALPS, T1WI, FLAIR), PET (Aβ, Tau, FDG), CSF biomarkers	ADNI cohort: 3 T MRI (T1, FLAIR, DTI; Taoka’s ALPS pipeline); DTI: 55 slices, voxel 2 × 2 × 2 mm^3^; ROIs on projection and association fibers; PET: AV45/FBB (Aβ), AV1451 (Tau), FDG (glucose metabolism)	Bilateral projection (SCR, corona radiata) and association fibers (SLF) at LV body	ALPS index; cross-sectional + longitudinal; combined with Aβ PET SUVR, tau PET, FDG PET, CSF Aβ42/pTau/tTau, hippocampal and AD-signature ROI volumes	Stepwise ↓ ALPS (CN > MCI > AD); lower ALPS predicted ↑ Aβ, brain atrophy, and clinical progression, mediated by Aβ and atrophy.
Chen et al. (2025)	MRI (DTI-ALPS, T1WI, T2WI, FLAIR), automated EPVS segmentation (VB-Net deep learning)	3 T GE Discovery MR750; DTI (*b* = 0, 1,000, 25 dirs, voxel = 0.75 × 0.75 × 1.5 mm^3^); 3D T1-MPRAGE (1 mm iso); preprocessing with FSL, ANTs, VB-Net	Basal ganglia (BG), centrum semiovale (CSO), midbrain (for EPVS); projection and association fibers near lateral ventricles (for ALPS)	ALPS index; EPVS volume and number; EPVS volume fraction (BG, CSO)	AD and aMCI had ↑ EPVS and ↓ ALPS vs. NC; ALPS correlated with cognition and combined EPVS+ALPS improved early AD discrimination.
Nakaya et al. (2025)	MRI (DTI-ALPS, T1WI, FLAIR), PET (Aβ-AV45), CSF biomarkers (Aβ42, T-tau, P-tau)	3 T ADNI-2 protocol; DTI for ALPS; volumetric T1 for choroid plexus and ventricles; FLAIR for WM lesions	Periventricular white matter (projection and association fibers for ALPS); choroid plexus; lateral ventricles	ALPS index; choroid plexus volume fraction (CPVF); lateral ventricular volume fraction (LVVF)	ALPS ↓ in Ab+ HC, SCD, MCI, AD vs. Ab– HC; higher CPVF/LVVF with severity; ALPS correlated with cognition, indicating early clearance impairment.
Jiao H. S. et al. (2025)	Simultaneous PET/MR (DTI-ALPS + early-phase Aβ PET, T1, FLAIR)	3 T PET/MR (United Imaging uPMR790); PET: ^18F-AV45, early-phase (0–5 min) for perfusion, late-phase (50–60 min) for Aβ; MRI: DTI (64 dirs, *b* = 1,000), 3D-MPRAGE, T2-FLAIR	Bilateral hippocampus, parahippocampus, caudate, thalamus (perfusion/ALPS); projection and association fibers near LV body for ALPS; hippocampus, WM, BG for PVS	ALPS index; early-phase Aβ SUVr (perfusion); baseline Aβ burden (CL); PVS fraction; WMH volume	AD and PAD had ↓ ALPS and ↓ perfusion vs. HC; ALPS correlated with perfusion and cognition; mediation showed perfusion deficits → ALPS decline → cognitive impairment.
Guo et al. (2025)	MRI (DTI-ALPS, 3D T1WI), CSF and plasma biomarkers (Aβ42, Aβ40, pTau)	ADNI cohort: 3 T GE scanner, DTI (41 dirs, *b* = 1,000), 3D T1 (1 × 1 × 1.2 mm^3^); CTPCS-HEAD cohort: 3 T Siemens Prisma, DTI (64 dirs, *b* = 1,000), 3D T1 (1 mm iso)	Projection and association fibers (atlas-based ROIs, FA > 0.3 to exclude WM impairment)	ALPS index; diffusion metrics (Dx, Dy, Dz); GMV ratio	ALPS ↓ in AD vs. CN/SMC/MCI (two cohorts); correlated with cognition and Aβ/pTau; lower baseline ALPS predicted MCI → AD conversion and SVM model showed good diagnostic accuracy.
Shang et al. (2024)	MRI (DTI-ALPS, T1WI, T2WI, FLAIR), fMRI, cognitive and sleep assessments	3 T Siemens Skyra; NODDI sequence (*b* = 0, 1,000, 2,000; 64 dirs); voxel 2 × 2 × 2 mm^3^; T1/T2/FLAIR; preprocessing with DSI Studio (QSDR, motion/eddy correction)	Projection fibers (SCR) and association fibers (SLF) bilaterally at LV body level	ALPS index; MMSE, MoCA; PSQI (sleep quality)	ALPS ↓ in AD with/without sleep disorder vs. NC, lowest in ADSD; ALPS linked to cognition and sleep quality; mediation showed sleep disorder → ALPS ↓ → cognitive decline; ROC cut-off gave good discrimination.
Sun et al. (2024)	MRI (DTI-ALPS, resting-state fMRI, T1WI)	3 T Philips Ingenia Elition; DTI: 64 dirs, *b* = 1,000, TR/TE = 3,950/96 ms, voxel 2 mm; fMRI: TR/TE = 1,000/30 ms, 480 volumes, 3.5 mm slices; 3D T1: TR/TE = 8.1/3.7 ms, 1 mm iso	Superior corona radiata (SCR) and superior longitudinal fasciculus (SLF) near LV body (for ALPS); whole-brain and 7 subnetworks (SMN, VAN, DAN, VN, LN, FPN, DMN) for SC-FC coupling	ALPS index (left, right, mean); SC-FC coupling values (whole-brain, subnetworks)	MCI showed left-sided ALPS ↓ and ↓ SC-FC coupling vs. HC; ALPS correlated with cognition and coupling; ROC demonstrated high diagnostic accuracy, highlighting left-hemisphere glymphatic impairment.
Sacchi et al. (2024)	MRI (DTI-ALPS, DKI, free-water DTI, T1, FLAIR) + CSF biomarker (AQP4)	3 T Philips Achieva; Multi-shell DWI (*b* = 1,000/2,000, 32 dirs each, 2.5 mm slices); T1 MPRAGE (1 mm iso), FLAIR (1 mm iso)	PVS in basal ganglia (BG) and centrum semiovale (CSO); Deep medullary veins (projection and association fibers near LV)	ALPS index (mean, L, R); Free-water WM (FW-WM); Mean kurtosis WM (MK-WM); PVS counts and PVSVF; CSF AQP4 concentration	AD had ↑ CSF-AQP4, ↑ FW-WM, ↓ ALPS, and ↑ PVS burden; changes stronger in AD than nAD; MCI showed variable ALPS linked to atrophy; results suggest AQP4/FW = early dysfunction, ALPS/MK/PVS = later-stage damage.
Hsu et al. (2023)	MRI (DTI-ALPS, structural T1w), PET (Aβ [18F-AV-45], Tau [18F-APN1607])	3 T Siemens PET/MRI; DTI (64 dirs, *b* = 1,000 s/mm^2^); T1-MPRAGE, FLAIR; PET standard protocols	Periventricular projection fibers (superior/posterior corona radiata), SLF, precuneus, frontal, parietal, temporal regions	ALPS index; amyloid SUVR; tau SUVR; GMV ratio	AD showed ↓ ALPS vs. NC; ALPS correlated with cognition and GM volume, and mediated the effect of Aβ/tau burden on cognitive decline.
Liang et al. (2023)	ALPS index (left, right, mean); SC-FC coupling values (whole-brain, subnetworks)	3 T GE Signa; DTI (TR/TE = 15,800/77 ms, voxel 2 × 2 × 2.5 mm^3^, *b* = 1,000, 55 slices, NEX = 3); processing with dTV. II.13 k software	Periventricular WM: projection fibers and superior longitudinal fasciculus (left hemisphere)	ALPS index (diffusivity ratio of Dx/Dy/Dz)	AD, MCI, VCI all had ↓ ALPS vs. NC; VCI > AD suggested drainage disorder; ALPS correlated with cognition, showing distinct mechanisms between AD and VCI.
Ota et al. (2022)	MRI (DTI-ALPS, T1WI), PET (Aβ: ^11C-PiB, Tau/Inflammation: ^18F-THK5351)	3 T Siemens Verio; DTI: 30 dirs, *b* = 1,000, TR/TE = 17,700/93 ms, slice 2 mm; 3D T1: TR/TE = 1,900/2.5 ms, voxel 0.98 mm	Projection and association fibers adjacent to lateral ventricle body (bilateral)	ALPS index (Dx/Dy/Dz ratio); global SUVR of ^11C-PiB and ^18F-THK5351	AD showed ↓ ALPS vs. HC; ALPS correlated positively with cognition, negatively with Aβ/Tau PET SUVR, indicating impaired clearance contributes to pathology.
Zhang et al. (2025)	MRI (DTI-ALPS, T1WI); PET: [^18F]Florbetapir (Aβ), [^18F]PI-2620 (tau), [^18F]FDG (neurodegeneration)	3 T uPMR790 PET/MRI (United Imaging Healthcare); DTI: 32 dirs, *b* = 1,000, TR = 4,699 ms, TE = 84 ms, voxel 1.95 × 1.95 × 4 mm^3^; 3D T1: 1 mm iso; PET tracers per GMP, standard uptake time and OSEM reconstruction	Bilateral SCR and SLF at LV body level (manual ROIs by neuroradiologists)	ALPS index; Aβ-PET SUVR; tau-PET SUVR; FDG-PET hypometabolism score	Stepwise ↓ ALPS (SCD > MCI > AD); ALPS linked to Aβ, tau, FDG, cognition; mediation showed Aβ (not tau) fully explained ALPS–cognition relationship, with stronger effect in EOAD.
Kim et al. (2024)	MRI (DTI, structural MRI, FLAIR, SWI), Amyloid PET	3 T Philips Ingenia; DTI (*b* = 0, 1,000 s/mm^2^, 32 dirs); 3D T1WI, 3D FLAIR, T2WI, SWI	Deep medullary veins near lateral ventricle body (projection, association, subcortical fibers)	ALPS index; Gray matter volumes (entorhinal, hippocampus, temporal pole, motor cortex); Amyloid SUVR	AD had ↓ ALPS vs. CN; ALPS correlated with cognition and GM volumes, but not with amyloid SUVR, suggesting independence from amyloid deposition.

Although all studies used MRI to derive the ALPS index, methodological differences remained evident (Table 2). A detailed summary of diffusion MRI acquisition parameters and preprocessing approaches is provided above. While the ALPS index was the primary outcome in nearly all studies, several investigations also included extended imaging metrics, such as perivascular space (PVS) volume (Chen et al., 2025; Sacchi et al., 2024; Jiao Y. et al., 2025; Kamagata et al., 2022; Zhang et al., 2024) or free-water fraction (FW) (Sacchi et al., 2024; Kamagata et al., 2022), and explored their associations with cognitive performance, PET imaging, or CSF biomarkers. Despite differences in imaging protocols and analytical approaches, the direction of findings was largely consistent across studies, with lower ALPS values observed in AD patients compared with controls (see Table 2).

### Meta-analysis

**Figure 2:**
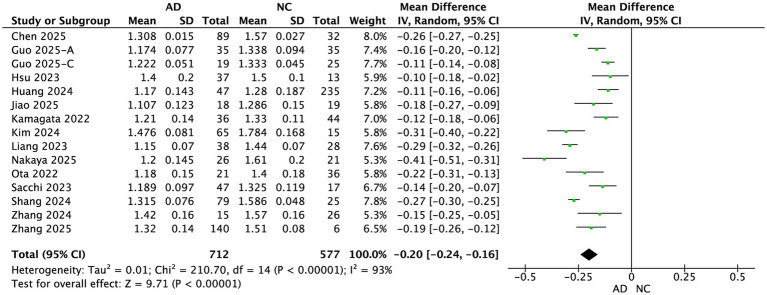
Forest plot of ALPS index in AD versus NC.

*AD vs NC*: Across 15 studies, pooled results showed that patients with AD had significantly lower ALPS values compared with NC (MD = −0.20, 95% CI –0.24 to −0.16, *p* < 0.00001) (Chen et al., 2025; Liang et al., 2023; Huang et al., 2024; Sacchi et al., 2024; Zhang et al., 2025; Jiao Y. et al., 2025; Kim et al., 2024; Shang et al., 2024; Hsu et al., 2023; Ota et al., 2022; Guo et al., 2025; Kamagata et al., 2022; Nakaya et al., 2025; Zhang et al., 2024). Heterogeneity was substantial (*I*^2^ = 93%, *p* < 0.00001). The direction of effect was consistent across studies, with confidence intervals not crossing zero. Larger studies (e.g., Chen et al., 2025; Shang et al., 2024) contributed more to the pooled estimate, while smaller studies (e.g., Nakaya et al., 2025; Zhang et al., 2024) showed similar trends with lower weights. Overall, these findings are consistent with reduced ALPS values in AD; however, given the high heterogeneity, the magnitude of the pooled effect should be interpreted with caution (see Figure 2: Main analysis).

*Subgroup analysis*: To explore sources of heterogeneity, subgroup analyses were conducted. Studies were categorized into clinical validation and mechanistic exploration subgroups based on their primary research objectives.

**Figure 3:**
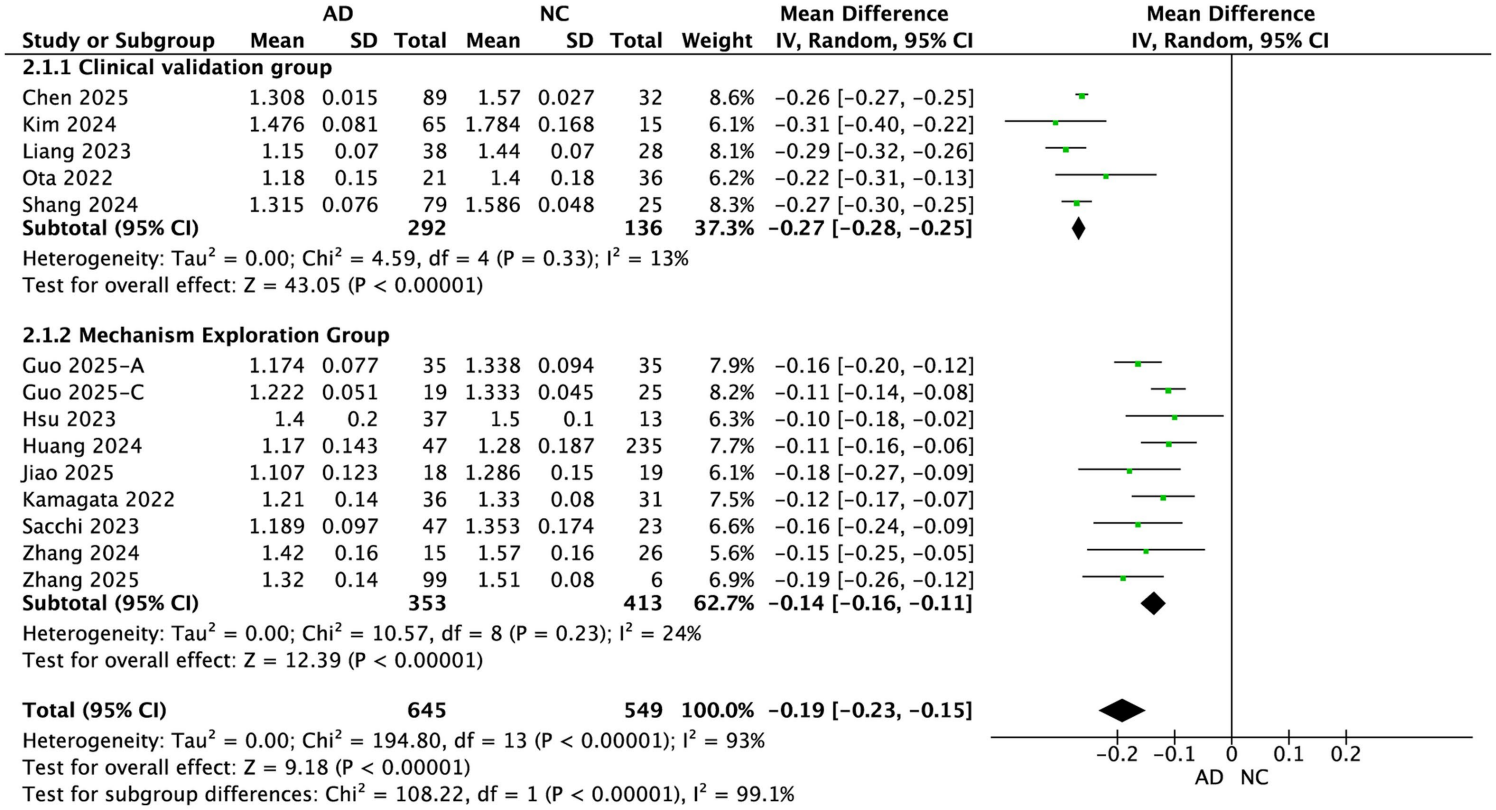
Subgroup analyses of ALPS index reduction in AD versus NC.

Clinical validation studies showed larger effect sizes (MD = −0.27, 95% CI –0.28 to −0.25) (Chen et al., 2025; Liang et al., 2023; Kim et al., 2024; Shang et al., 2024; Ota et al., 2022), with low heterogeneity (*I*^2^ = 13%, *p* = 0.33), and a fixed-effects model was applied (Figure 3). Mechanistic exploration studies showed smaller effect sizes (MD = −0.14, 95% CI –0.16 to −0.11) (Huang et al., 2024; Sacchi et al., 2024; Zhang et al., 2025; Jiao Y. et al., 2025; Hsu et al., 2023; Guo et al., 2025; Kamagata et al., 2022; Zhang et al., 2024), also with low heterogeneity (*I*^2^ = 24%, *p* = 0.27), and a fixed-effects model was used (Figure 3).

Both subgroups showed consistent directions of effect, supporting a reduction in ALPS values in AD (Figure 3). Sensitivity analysis indicated that one study substantially contributed to heterogeneity (Nakaya et al., 2025). Exclusion of this study markedly reduced *I*^2^ without changing the effect size or statistical significance (see Supplementary Figure S1).

Other subgrouping strategies (e.g., based on imaging protocols or diagnostic criteria) may also be informative; however, the present classification was chosen to reflect differences in study intent and analytical focus.

**Figure 4:**
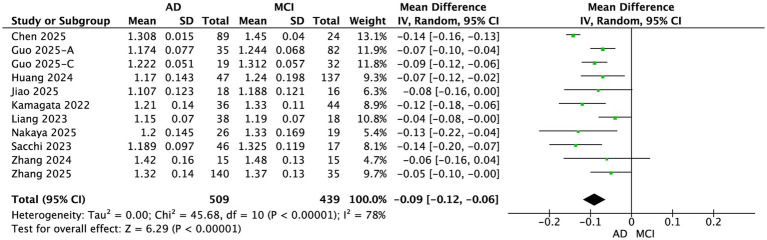
Forest plot of ALPS index in AD versus MCI.

*AD vs MCI*: Pooled results from 11 studies showed that patients with AD had significantly lower ALPS values than those with MCI (MD = −0.09, 95% CI –0.12 to −0.06, *p* < 0.00001) (Chen et al., 2025; Liang et al., 2023; Huang et al., 2024; Sacchi et al., 2024; Zhang et al., 2025; Jiao Y. et al., 2025; Guo et al., 2025; Kamagata et al., 2022; Nakaya et al., 2025; Zhang et al., 2024). Heterogeneity was moderate to high (*I*^2^ = 78%), although confidence intervals consistently excluded zero. Sensitivity analysis showed that exclusion of Chen et al. (2025) reduced heterogeneity to 28%, while the pooled effect size and statistical significance remained unchanged (MD = −0.08, 95% CI –0.09 to −0.06), suggesting that the overall findings were relatively robust despite study-level variability (see Figure 4: AD vs. MCI and Supplementary Figure S2).

*MCI vs NC (Supplementary analysis)*: MCI patients also showed lower ALPS values compared with NC (MD = −0.11, 95% CI –0.15 to −0.06), although heterogeneity remained high (*I*^2^ = 92%). These results should be interpreted with caution, and detailed findings are presented in Supplementary Figure S3.

*Publication Bias*: Funnel plot analyses were performed to assess potential publication bias (Supplementary Figures S4, S5). The plots for the overall comparison (AD vs. NC), subgroup analyses (clinical validation and mechanistic exploration groups), and sensitivity analyses appeared largely symmetrical, suggesting no obvious publication bias. However, the reliability of publication bias assessment in this meta-analysis is limited by the relatively small number of included studies in certain comparisons. Funnel plot symmetry should therefore be interpreted with caution, as the statistical power to detect asymmetry is reduced in small meta-analyses.

*Certainty of evidence (GRADE assessment)*: The certainty of evidence for all three main comparisons (AD vs. NC, AD vs. MCI, and MCI vs. NC) was rated as low. This primarily reflects the observational nature of the included studies and the presence of substantial heterogeneity, particularly in the AD vs. NC and MCI vs. NC analyses. No major concerns were identified regarding indirectness, imprecision, or publication bias.

Overall, the findings suggest a stepwise reduction in ALPS values across the AD continuum; however, given the observational design and substantial heterogeneity, these results should be interpreted with caution, and further well-designed longitudinal studies are needed to strengthen the evidence base.

## Discussion

Our meta-analysis suggests a stepwise reduction in ALPS values across the AD continuum, from cognitively normal individuals to MCI and Alzheimer’s disease. This pattern may suggest that alterations in glymphatic-related processes emerge early and progress with disease severity, in line with previous hypotheses that impaired waste clearance may precede overt amyloid and tau pathology.

Importantly, although the direction of effects was generally consistent across studies, substantial between-study heterogeneity was observed. This heterogeneity requires careful interpretation and is discussed in detail below.

### Main findings and heterogeneity

The substantial heterogeneity observed across analyses likely reflects a combination of methodological variability and underlying biological differences, both of which should be considered when interpreting the pooled estimates.

From a methodological perspective, variability in diffusion MRI acquisition parameters—such as b-values, number of gradient directions, and spatial resolution—may influence sensitivity to water diffusion along perivascular spaces, thereby affecting ALPS measurements and introducing systematic differences across studies. In addition, differences in ROI placement and measurement protocols likely represent a major source of variability. Because the ALPS index is derived from directional diffusivity within specific periventricular white matter regions, even subtle differences in ROI positioning may alter the relative contributions of projection fibers, association fibers, and adjacent perivascular structures.

Notably, while most studies restricted ROIs to projection and association fibers adjacent to the lateral ventricles, others extended analyses to regions such as the basal ganglia (Li W. X. et al., 2024). This difference is not merely technical, as perivascular changes in the basal ganglia may be more closely related to disease-associated pathology. Previous studies have reported a negative association between ALPS values and enlarged perivascular spaces in the basal ganglia (BG-ePVS; Liu et al., 2021), and both reduced ALPS values and increased PVS burden have been associated with small vessel disease progression (Li H. et al., 2024).

Therefore, inclusion of different anatomical regions may improve sensitivity to glymphatic-related alterations, but may also introduce systematic variability rather than random noise, thereby reducing comparability across studies. Additional methodological variability may arise from differences in preprocessing pipelines, including motion correction, distortion correction, and tensor fitting, all of which may influence diffusion signal estimation. Furthermore, factors such as partial volume effects near the ventricles, crossing fiber configurations, and age-related white matter changes may further confound ALPS measurements.

From a biological perspective, heterogeneity may reflect differences in disease stage, diagnostic criteria, and participant characteristics across studies. The inclusion of diverse populations—including cognitively normal individuals, amyloid-defined subgroups, subjective cognitive decline, MCI, and AD—may introduce variability in both baseline and disease-related glymphatic function. In particular, differences in control group definitions (e.g., cognitively normal versus amyloid-negative individuals) may influence baseline reference levels and, consequently, the magnitude of group differences, especially in early or preclinical stages.

Importantly, methodological and biological sources of heterogeneity are likely intertwined. While methodological differences may introduce systematic variation, biological variability may reflect genuine differences in disease progression. Accordingly, the high *I*^2^ values should be interpreted in the context of both methodological diversity and the inherent complexity of glymphatic alterations in AD, rather than being viewed solely as a limitation.

Despite these sources of heterogeneity, the direction of effect was generally consistent across studies, with a progressive decrease in ALPS values along the AD continuum. This consistency supports the overall pattern of findings; however, the magnitude of the pooled effect should be interpreted with caution.

### Potential biological implications

The observed gradient in ALPS values (NC > MCI > AD) is consistent with the hypothesis that alterations in glymphatic function may emerge early and progress with disease severity. Several biological mechanisms have been proposed to underlie these changes. For example, loss of aquaporin-4 (AQP4) polarization at astrocytic end-feet may impair fluid exchange (Iliff et al., 2012; Mestre et al., 2018), while age-related vascular stiffening may reduce perivascular pulsatility (Nedergaard and Goldman, 2020). In addition, reactive astrogliosis has been suggested to disrupt interstitial fluid dynamics (Da Mesquita et al., 2018). Together, these processes may contribute to reduced efficiency of solute clearance in the brain.

Impaired clearance of metabolic waste, including amyloid-*β* and tau, has been hypothesized to contribute to neurodegeneration. Conversely, accumulation of these proteins may further alter perivascular pathways and tissue properties, suggesting a potentially bidirectional relationship between protein aggregation and clearance dysfunction.

However, it is important to emphasize that the ALPS index is an indirect imaging marker derived from diffusion properties and does not directly measure glymphatic flow. Changes in ALPS values may also be influenced by other factors, including white matter degeneration, vascular alterations, and age-related microstructural changes. Therefore, while the observed stepwise decrease in ALPS is consistent with proposed mechanisms of glymphatic dysfunction, it should be interpreted as supportive rather than definitive evidence.

### Clinical significance

From a clinical perspective, the ALPS index may provide complementary information to existing AD biomarkers (Taoka et al., 2024). While amyloid and tau markers primarily reflect pathological burden, ALPS is thought to reflect diffusion-based features that may be related to fluid transport and waste clearance in the brain (Buccellato et al., 2022). Considering these dimensions together raises the possibility of extending the current A/T/(N) framework to incorporate glymphatic function, forming a conceptual “A/T/(N)/(G)” model. Within such a framework, individuals who are positive for amyloid or tau and also show reduced ALPS values may represent a subgroup at potentially higher risk of disease progression and could be of interest for further investigation in future studies.

Another practical strength of ALPS is its accessibility. Unlike PET or CSF testing, it can be derived from conventional diffusion MRI, requires no invasive procedures, and is relatively low cost (Costa et al., 2024). These features make it suitable for repeated and longitudinal assessment in research settings. In addition, as strategies aimed at improving glymphatic function—including sleep-based interventions, non-invasive stimulation, and surgical approaches such as lymphatic–venous anastomosis—begin to be explored, ALPS may serve as a potential imaging outcome measure for tracking treatment-related changes (Kamagata et al., 2022; Fatima et al., 2025). However, these applications remain preliminary and require further validation.

Recent methodological work has also suggested ways to improve ALPS-based assessment. Radiomics may capture subtle microstructural features beyond conventional ROI-based approaches and may improve sensitivity to early glymphatic-related alterations (Li et al., 2020; Xia et al., 2024). Deep learning techniques may help standardize ROI placement, reduce operator dependence, and integrate multimodal MRI data. Early studies applying convolutional neural networks and machine learning pipelines have reported improved performance in distinguishing AD from controls compared with traditional approaches (Yuan et al., 2025; Jiang et al., 2022). These methods may further enhance the utility of ALPS by improving measurement consistency and supporting more individualized risk assessment; however, they remain at an early stage and require further validation before clinical translation (Aghajanian et al., 2025).

### Future directions

To better establish the clinical and biological relevance of ALPS, several directions should be pursued. First, large prospective and longitudinal studies are needed to determine whether alterations in ALPS precede or follow amyloid and tau accumulation—a distinction that is important for understanding its role in disease pathogenesis. Preliminary evidence suggests that reductions in ALPS may already be detectable in early stages such as subjective cognitive decline (Li Y. et al., 2024), but further validation is required. Second, standardization of MRI acquisition and analysis protocols remains critical. Variability in diffusion parameters and ROI placement can substantially affect ALPS measurements, and recent studies suggest that more standardized and bilateral ROI approaches may improve reproducibility (Ulloa et al., 2025; Taoka et al., 2022). Harmonization across centers will be important for enabling reliable comparisons and may help support future clinical applications. Third, ALPS should be evaluated alongside established biomarkers. Multimodal approaches combining ALPS with CSF Aβ42/40, plasma p-tau, PET imaging, or structural MRI markers may provide a more comprehensive view of disease progression and may improve staging accuracy (Yu et al., 2025). Finally, future interventional studies targeting glymphatic function could consider incorporating ALPS as an exploratory outcome measure. Although absolute values may vary across scanners, within-subject longitudinal changes may offer useful insights into treatment effects.

### Limitations

Several limitations of this meta-analysis should be acknowledged. First, all included studies were observational and predominantly cross-sectional, which limits causal inference regarding the relationship between ALPS alterations and disease progression. Second, substantial methodological heterogeneity existed across studies, particularly in diffusion MRI acquisition parameters, ROI definitions, and preprocessing pipelines, which may affect the comparability of ALPS measurements. Third, clinical heterogeneity was evident among included populations, with variability in diagnostic criteria, disease stages, and control group definitions, including cognitively normal and amyloid-negative individuals. Such differences may influence baseline values and contribute to between-study variability. Fourth, the ALPS index is an indirect imaging marker derived from diffusion properties and does not directly measure glymphatic flow. Therefore, changes in ALPS values should be interpreted with caution and may be influenced by other factors, such as white matter degeneration, vascular changes, and age-related microstructural alterations. Finally, although no obvious publication bias was detected, the relatively small number of studies included in some analyses limits the reliability of bias assessment.

## Data Availability

The original contributions presented in the study are included in the article/Supplementary material, further inquiries can be directed to the corresponding author.

## Supplementary material

SUPPLEMENTARY FIGURE S1 Subgroup analysis including Nakaya et al. (2025).

SUPPLEMENTARY FIGURE S2 Sensitivity analysis of AD versus MCI after excluding Chen et al. (2025).

SUPPLEMENTARY FIGURE S3 Forest plot of ALPS index in MCI versus NC.

SUPPLEMENTARY FIGURE S4 Funnel plot of the 15 included studies.

SUPPLEMENTARY FIGURE S5 Funnel plot of subgroup analysis after excluding Nakaya et al. (2025).

SUPPLEMENTARY TABLE S1 AHRQ risk-of-bias assessment table.
