# Supplementary figures and images for: Imaging biomarkers of glymphatic system dysfunction in Alzheimer’s disease: a systematic review and meta-analysis with a focus on DTI-ALPS index
Source: Front Aging Neurosci. 2026 Jun 1;18:1749316. doi: 10.3389/fnagi.2026.1749316 (PMC13266463; doi:10.3389/fnagi.2026.1749316)

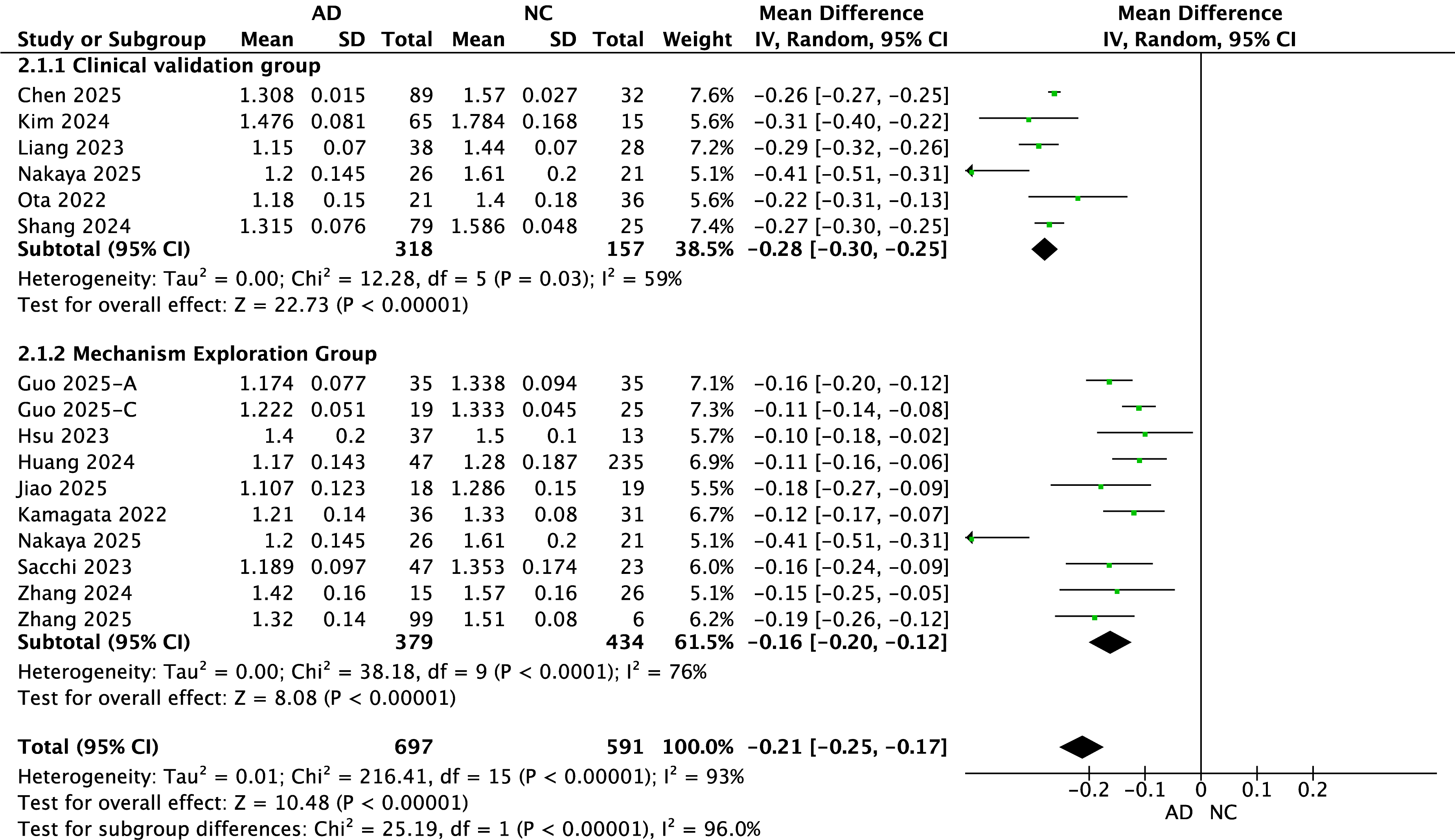

Supplement: SUPPLEMENTARY FIGURE S1 — Subgroup analysis including Nakaya et al. (2025). [file Image_1.TIF]

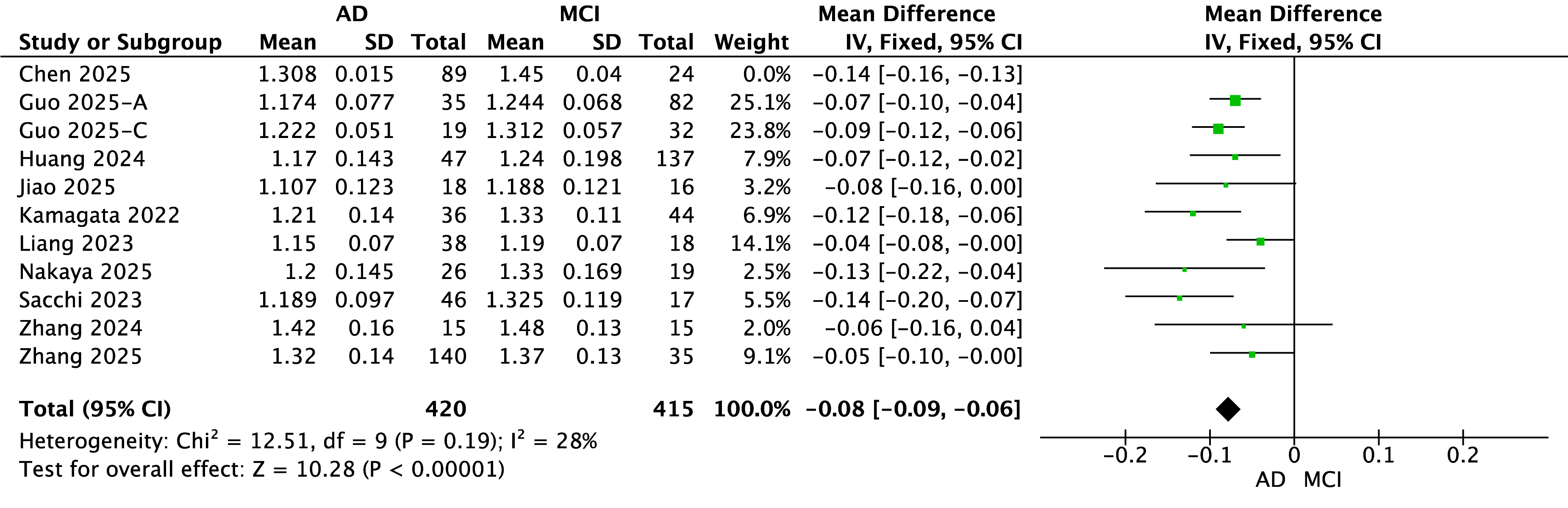

Supplement: SUPPLEMENTARY FIGURE S2 — Sensitivity analysis of AD versus MCI after excluding Chen et al. (2025). [file Image_2.TIF]

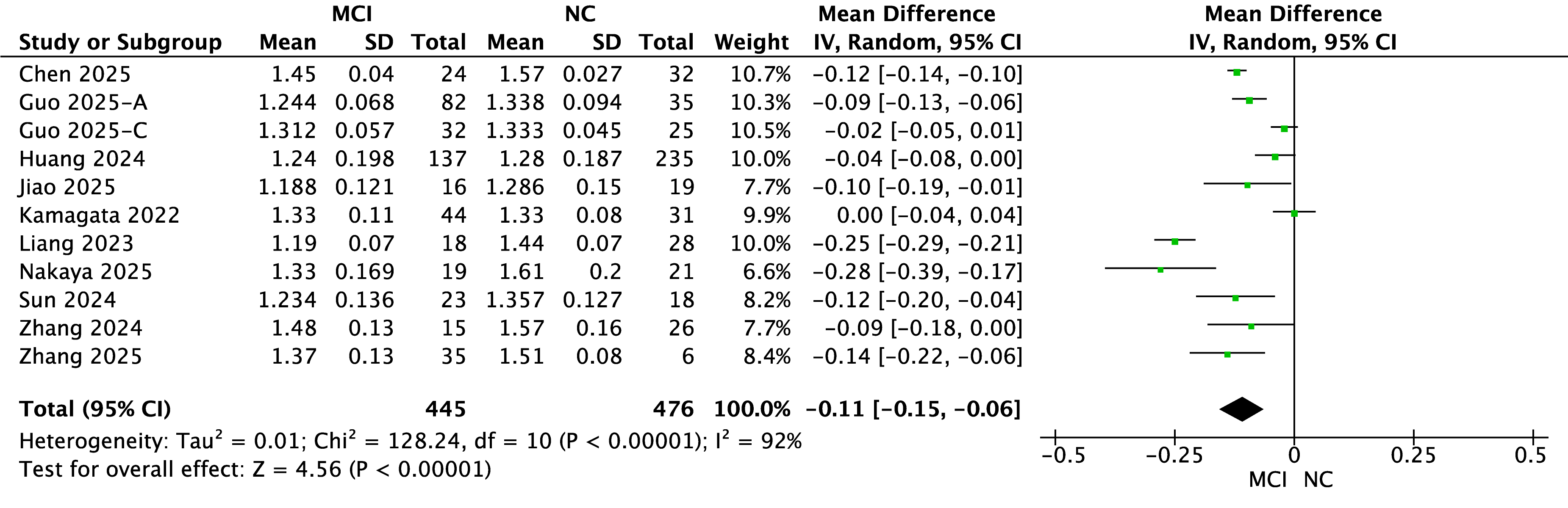

Supplement: SUPPLEMENTARY FIGURE S3 — Forest plot of ALPS index in MCI versus NC. [file Image_3.TIF]

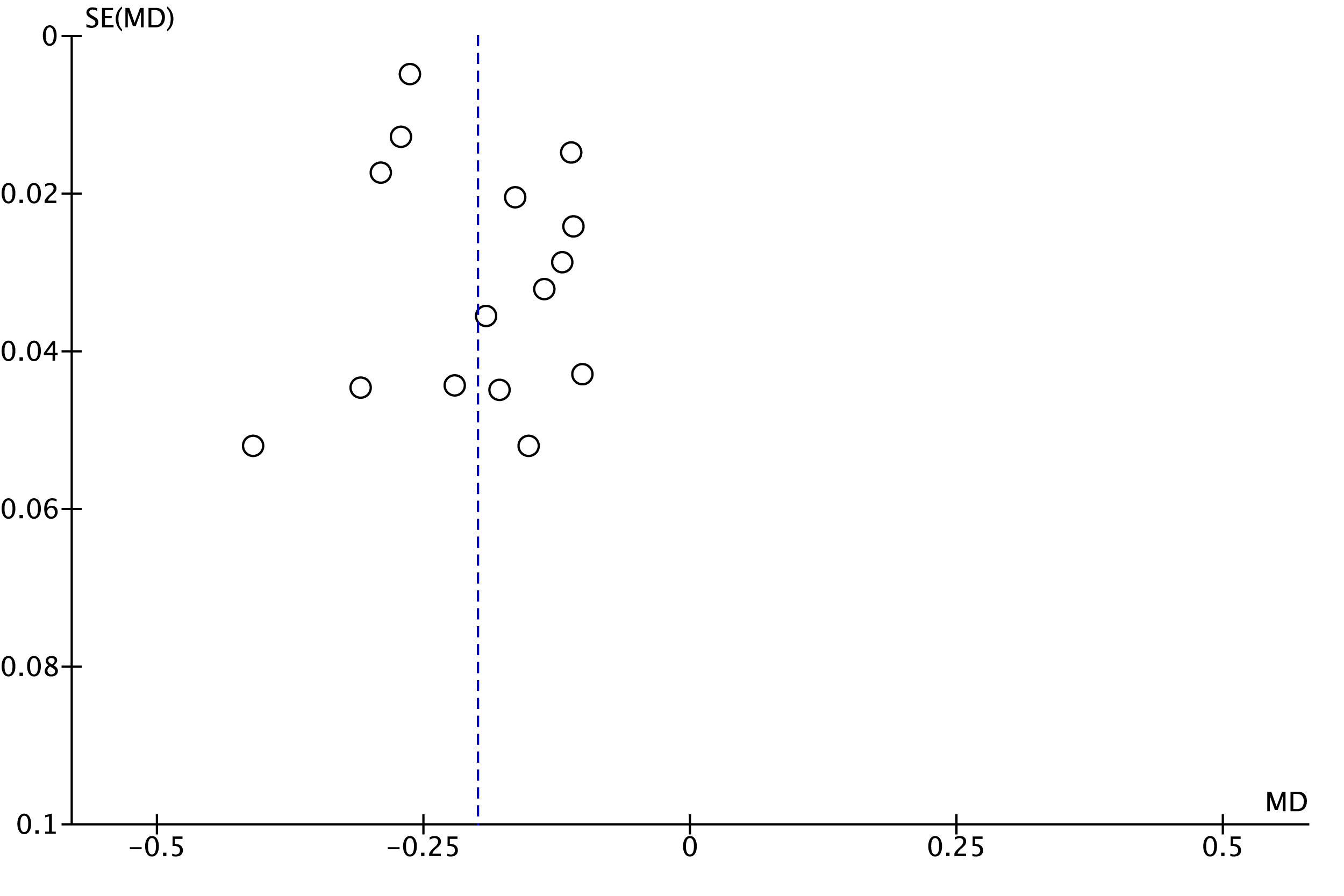

Supplement: SUPPLEMENTARY FIGURE S4 — Funnel plot of the 15 included studies. [file Image_4.TIF]

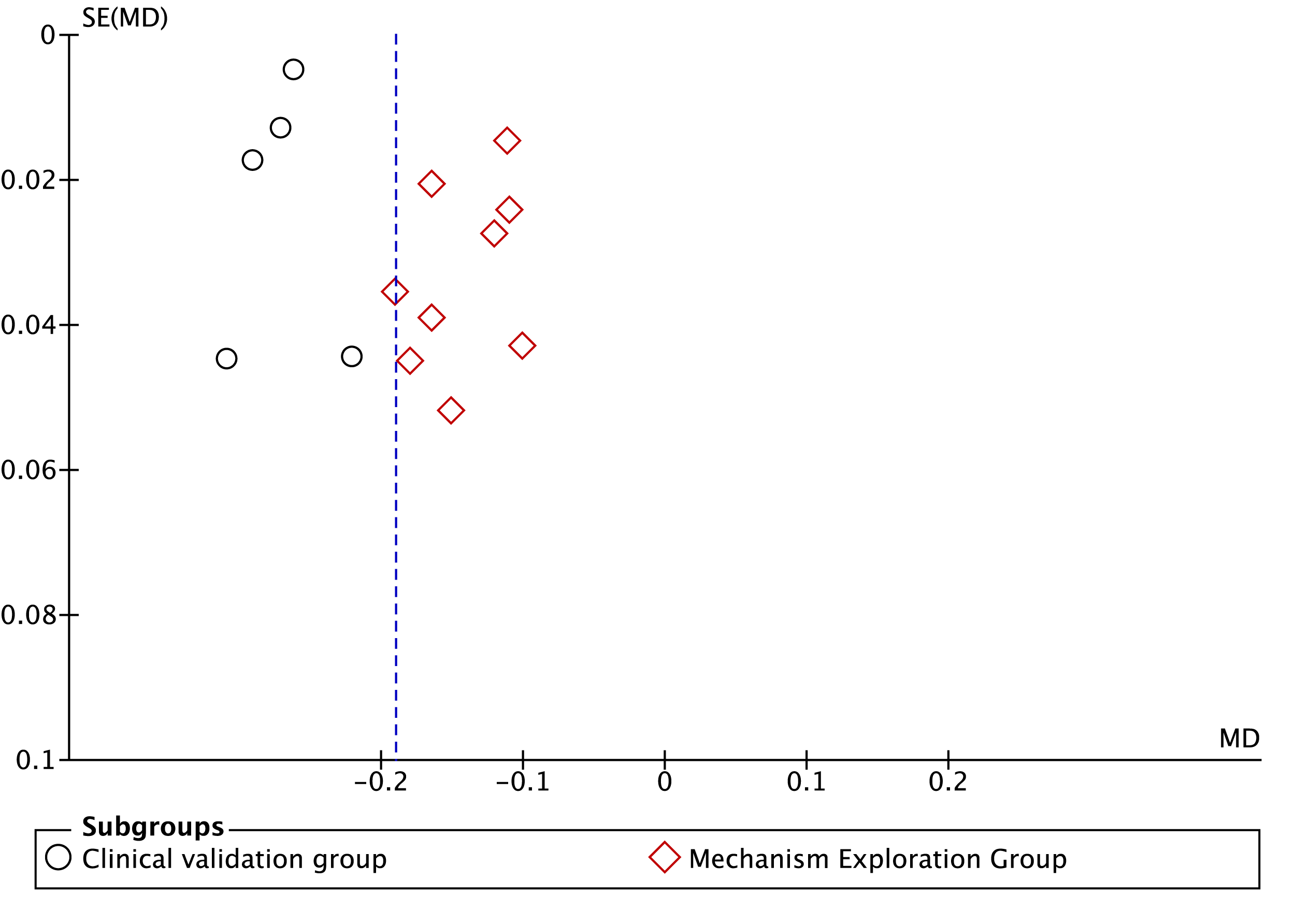

Supplement: SUPPLEMENTARY FIGURE S5 — Funnel plot of subgroup analysis after excluding Nakaya et al. (2025). [file Image_5.TIF]
