# Supplementary material for: Imaging biomarkers of glymphatic system dysfunction in Alzheimer’s disease: a systematic review and meta-analysis with a focus on DTI-ALPS index
Source: Front Aging Neurosci. 2026 Jun 1;18:1749316. doi: 10.3389/fnagi.2026.1749316 (PMC13266463; doi:10.3389/fnagi.2026.1749316)
Supplement: SUPPLEMENTARY TABLE S1 — AHRQ risk-of-bias assessment table. [file Table_1.docx]

| **Study (First author, Year)** | **Source clearly stated** | **Inclusion/exclusion defined** | **Time period given** | **Consecutive subjects** | **Characteristics described** | **Exposure measured** | **Outcome measured** | **Confounders considered** | **Handling of missing data** | **Response rate/Completeness** | **Follow-up explained** | **AHRQ score** |
| --- | --- | --- | --- | --- | --- | --- | --- | --- | --- | --- | --- | --- |
| Zhang (2024) |  |  |  |  |  |  |  |  |  |  |  | 8 |
| Kamagata (2022) |  |  |  |  |  |  |  |  |  |  |  | 8 |
| Huang (2024) |  |  |  |  |  |  |  |  |  |  |  | 9 |
| Chen (2025) |  |  |  |  |  |  |  |  |  |  |  | 8 |
| Nakaya (2025) |  |  |  |  |  |  |  |  |  |  |  | 6 |
| Jiao (2025) |  |  |  |  |  |  |  |  |  |  |  | 8 |
| Guo (2025) |  |  |  |  |  |  |  |  |  |  |  | 8 |
| Shang (2024) |  |  |  |  |  |  |  |  |  |  |  | 8 |
| Sun (2024) |  |  |  |  |  |  |  |  |  |  |  | 7 |
| Sacchi (2023) |  |  |  |  |  |  |  |  |  |  |  | 9 |
| Hsu (2023) |  |  |  |  |  |  |  |  |  |  |  | 8 |
| Liang (2023) |  |  |  |  |  |  |  |  |  |  |  | 8 |
| Ota (2022) |  |  |  |  |  |  |  |  |  |  |  | 8 |
| Zhang (2025) |  |  |  |  |  |  |  |  |  |  |  | 9 |
| Kim (2024) |  |  |  |  |  |  |  |  |  |  |  | 8 |

|  | YES |
| --- | --- |
|  | N/A |
|  | NO |
